# MultiGATAE: A Novel Cancer Subtype Identification Method Based on Multi-Omics and Attention Mechanism

**DOI:** 10.3389/fgene.2022.855629

**Published:** 2022-03-21

**Authors:** Ge Zhang, Zhen Peng, Chaokun Yan, Jianlin Wang, Junwei Luo, Huimin Luo

**Affiliations:** ^1^ School of Computer and Information Engineering, Henan University, Kaifeng, China; ^2^ College of Computer Science and Technology, Henan Polytechnic University, Jiaozuo, China

**Keywords:** cancer subtype identification, multi-omics, graph attention network, omics-level attention mechanism, cluster

## Abstract

Cancer is one of the leading causes of death worldwide, which brings an urgent need for its effective treatment. However, cancer is highly heterogeneous, meaning that one cancer can be divided into several subtypes with distinct pathogenesis and outcomes. This is considered as the main problem which limits the precision treatment of cancer. Thus, cancer subtypes identification is of great importance for cancer diagnosis and treatment. In this work, we propose a deep learning method which is based on multi-omics and attention mechanism to effectively identify cancer subtypes. We first used similarity network fusion to integrate multi-omics data to construct a similarity graph. Then, the similarity graph and the feature matrix of the patient are input into a graph autoencoder composed of a graph attention network and omics-level attention mechanism to learn embedding representation. The K-means clustering method is applied to the embedding representation to identify cancer subtypes. The experiment on eight TCGA datasets confirmed that our proposed method performs better for cancer subtypes identification when compared with the other state-of-the-art methods. The source codes of our method are available at https://github.com/kataomoi7/multiGATAE.

## 1 Introduction

Cancer is one of the leading causes of death worldwide and is a serious threat to human health (Sung et al., 2021). Cancer is extremely heterogeneous, and distinct molecular subtypes have different clinical outcomes (Zhao and Yan, 2019). The goal of cancer subtype identification is to discover patient groups with different clinical outcomes, thus facilitating personalized treatment (Liang et al., 2021). For instance, four potential molecular subtypes of gastric cancer, i.e., EBV, MSI, GS, and CIN, were uncovered by The Cancer Genome Atlas (TCGA) project (Bass et al., 2014), and each of these four molecular subtypes has specific clinical significance signatures (Sohn et al., 2017). Therefore, cancer subtype identification is of great importance.

The rapid development of high throughput sequencing technology has made a massive amount of omics data from the different levels available. This provides an opportunity to investigate the heterogeneity of cancer and to identify cancer subtypes (Zhao et al., 2019). Since omics data lack labels associated with cancer subtypes, cancer subtype identification is usually addressed using clustering (Xu et al., 2019). Earlier studies usually used only single-omics data; however, single-omics data provide only a very limited view on cancer subtype identification (Gomez-Cabrero et al., 2014; Le Van et al., 2016). Thus, many researchers integrate multi-omics data to identify cancer subtypes. Yang et al. (2021a) proposed a computational method called Deep Subspace Mutual Learning (DSML). DSML constructed branching models for each type of omics data and then constructed a main stem model to optimize the feature representation learned from single-omics data. Finally, spectral clustering was applied to the learned representation to identify cancer subtypes. Chaudhary et al. (2018) applied an autoencoder to process multi-omics data to gain low-dimensional features, then the features were further filtered using Cox-PH analysis. Finally, K-means was applied to the resulting features to cluster cancer subtypes. While using multi-omics data provides a comprehensive view, it also introduces additional computational costs.

Apart from the differences in the used data, some studies have typically focused on analyzing the features of omics data and the distribution of each data type to identify cancer subtypes. Shen et al. (2009) proposed an integrative clustering method named iCluster. iCluster models the subtypes of cancer as latent variables which can be simultaneously estimated from the omics data. Yang et al. (2021) introduced a deep-learning method named Subtype-GAN for cancer subtyping. Subtype-GAN consists of three modules: encoder, decoder, and discriminator. The encoder takes multi-omics data as input and encodes them into low-dimensional representation. The decoder reconstructs the original input using the low-dimensional representation. The discriminator is used to force the representation encoded by the encoder to follow the prior Gaussian distribution. Finally, Consensus GMM clustering is applied to the low-dimensional representation to determine the most appropriate clustering number and to predict the subtype results. However, these methods are limited by strong assumptions on the distribution of the omics data (Song et al., 2021). Noise in the omics data may affect the results of cancer subtyping. Similarity-based approaches for multi-omics data can avoid this problem (Song et al., 2021). Wang et al. (2014) proposed a method named Similarity Network Fusion (SNF) for integrating multi-omics data. SNF first generates a sample similarity network for each type of data and then iteratively fuses these similarity networks. Zhao and Yan (2019) proposed a cancer subtyping method named Molecular and Clinical Networks Fusion (MCNF), which integrates multi-omics and clinical data. MCNF first applies unsupervised random forest to multi-omics and clinical data to generate a patient affinity network and then uses random walk to fuse the patient affinity networks. After obtaining the fused network, PAM clustering is used to identify the cancer subtypes. Yang et al. (2021b) introduced a clustering method, Deep Subspace Fusion Clustering (DSFC), for cancer subtype prediction. DSFC calculates data self-expressiveness to generate a patient similarity network, and then fuses these patient similarity networks to gain a combined network. Finally, spectral clustering is performed on the combined similarity network to find cancer subtypes. Similarity-based approaches usually just use the omics data to generate a similarity network, and completely disregard the feature information of the omics data in subsequent calculations. This may lead to incomplete subtype results.

To make full use of the feature information of the omics data and the similarity graph, a graph-based neural network was used because it takes both the feature information as well as the similarity graph into consideration (Wu et al., 2021). In this work, we proposed a deep-learning method named multiGATAE for cancer subtype identification. multiGATAE first applies multi-omics data to construct a similarity graph and then establish a graph autoencoder network which is composed of a graph attention network and an omics-level attention mechanism to obtain the embedding representation. Finally, the K-means clustering method is applied to the embedding representation to identify cancer subtypes. multiGATAE was compared with serval state-of-the-art methods on eight public cancer datasets, and the results demonstrated that our proposed method performs better.

The remainder of this article is organized as follows. In section 2, we present the proposed method. The datasets we used and the experiment results are shown in section 3. In section 4, we conclude this article and discuss the future work.

## 2 Materials and Methods

**FIGURE 1:**
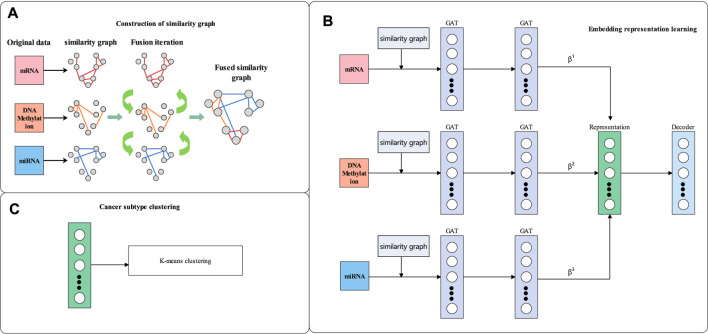
Workflow of multiGATAE. **(A)** Construction of similarity graph. **(B)** Embedding representation learning. **(C)** Cancer subtype clustering.

In this section, the details of our proposed-method multiGATAE are described. Our proposed method consists of three parts. Firstly, a similarity graph is constructed by integrating multi-omics data. Then, the similarity graph and omics data are input to a graph autoencoder composed of a graph attention network and omics-level attention mechanism to learn the embedding representation. Finally, the K-means method is applied to the embedding representation to identify the cancer subtypes. The workflow of multiGATAE is shown in Figure 1.

### 2.1 Construction of Similarity Graph

A network fusion method named SNF (Wang et al., 2014) was used to construct the similarity graph. SNF first generated specific similarity graphs for each omics, and then iteratively integrated them to construct the combined similarity graph. Suppose that there are n patients and m views (such as mRNA, miRNA, and DNA methylation). The similarity graph is defined as a graph G = (V, E), where V is the set of patients \{x_1_, x_2_, x_3_ … , x_*n*_\} and the edges E correspond to the similarity between vertices v ∈ V. The edge weights are represented by an n × n similarity matrix W, and W is computed by Eq. 1.

$$W_{i,j}=\exp\left(-\frac{\phi^{2}\left(x_{i},x_{j}\right)}{\alpha \gamma_{i,j}}\right)$$ (1)

where *α* is a hyperparameter, *ϕ* (x_*i*_, x_*j*_) is the Euclidean distance between patients x_*i,*_ and x_*j*_, and *γ*_*i*,*j*_ is used to eliminate the scaling problem. In order to compute the fused matrix from multiple types of data, the similarity matrix is normalized as Eq. 2.

$$P_{i,j}=\left\{\begin{array}{cc} \frac{W_{i,j}}{2\underset{k\neq i}{\sum }W_{i,k}}\; & j\neq i\\ \frac{1}{2}\; & j=i \end{array}\right.$$ (2)

assuming N_*i*_ is a set of x_*i*_ 's neighbors. Then, the local affinity matrix S is calculated by Eq. 3.

$$S_{i,j}=\left\{\begin{array}{cc} \frac{W_{i,j}}{\underset{k\in N_{i}}{\sum }W_{j,k}}\; & j\in N_{i}\\ 0\; & otherwise \end{array}\right.$$ (3)

Let P_*t*_^(*h*)^ represent the normalized similarity matrix of h-th type data (1 ≤ h ≤ m) in the t-th iteration; P_*t*_^(*h*)^ is updated according to Eq. 4.

$$P_{t+1}^{\left(h\right)}=S^{\left(h\right)}\left(\frac{\underset{k\neq h}{\sum }P_{t}^{\left(k\right)}}{m-1}\right){\left(S^{\left(h\right)}\right)}^{T}$$ (4)

where S^(*h*)^ represents the local affinity matrix of the h-th type data. Through this process of continuous iterative fusion, the combined similarity graph, which contains complementary information from three omics datasets, is finally obtained and then taken as the input of multiGATAE to learn the embedding representation.

### 2.2 Embedding Representation Learning

Cancer subtype identification is a typical clustering problem because of the lack of labels associated with the cancer subtypes (Xu et al., 2019). A key problem of clustering is how to capture the feature information of the nodes and the relationship between the nodes (Wang et al., 2019). A graph-based neural network may be able to solve this problem because it considers both the feature information of the nodes as well as the similarity relationships (Wu et al., 2021). In this work, we constructed a graph autoencoder composed of a graph attention network and omics-level attention mechanism to learn the embedding representation. We first introduce the Graph Convolutional Network (GCN) (Kipf and Welling, 2016a). The aim of the GCN is to learn a latent representation Z based on the node feature matrix X, which describes every node in the graph, and a similarity matrix A, which encodes the similarities between the nodes. The layer-wise propagation rule of GCN can be formulated as Eq. 5.

$$Z^{L}=\sigma \left({\tilde{D}}^{-\frac{1}{2}}\tilde{A}{\tilde{D}}^{-\frac{1}{2}}Z^{L-1}W^{L-1}\right)$$ (5)

where $\tilde{A}$ = A + E, which is a similarity matrix adding self-connections. $\tilde{D}$ is the diagonal node degree matrix of $\tilde{A}$ . *σ*(⋅) is a nonlinear activation function. *Z* ^*L*^ is the output of the L layer. However, a limitation of GCN is that it does not assign different weights to different nodes in the neighborhood (Veličković et al., 2017). In a practical situation, different neighbor nodes may play different roles for the current node. Therefore, we chose to use GAT (Veličković et al., 2017) which aggregates the neighbor nodes through the self-attention mechanism (Vaswani et al., 2017) and enables the adaptive assignment of weights to different neighbors. GAT first computes the attention coefficients by Eq. 6

$$e_{ij}=\alpha \left(Wx_{i},Wx_{j}\right)$$ (6)

where *α*(⋅) is a shared attentional mechanism, and *x*_*i*_ and *x*_*j*_ represent the features of node i and node j, respectively. The attention coefficients indicate the importance of node j's features to node i. To make the attention coefficients comparable across different nodes, the softmax function is used to normalize them:

$$\alpha_{ij}=softmax\left(e_{ij}\right)$$ (7)

The normalized attention coefficients are then used to compute the final output Z as Eq. 8

$$Z^{L}=\sigma \left(\alpha_{ij}{\tilde{D}}^{-\frac{1}{2}}\tilde{A}{\tilde{D}}^{-\frac{1}{2}}Z^{L-1}W^{L-1}\right)$$ (8)

In order to make the output Z more approximate to the similarity graph A, we propose an omics-level attention mechanism to aggregate the output of multi-omics. The attention score is defined as Eq. 9

$$w^{i}=v^{T}\tanh\left(W_{z}\cdot Z^{i}+W_{a}\cdot A\right)$$ (9)

where *w* ^*i*^ and *Z* ^*i*^ represent the attention score and the output of omics i. v, *W*_*z,*_, and *W*_*a*_ are trainable vectors. As mentioned above, we normalize the omics-level attention scores using the softmax function as Eq. 10

$$\beta^{i}=softmax\left(w^{i}\right)$$ (10)

We then obtain the final representation *Z* ^*final*^ by aggregating the output of multi-omics as Eq. 11.

$$Z^{\mathrm{fi}nal}=\sum \left(\beta^{i}Z^{i}\right)$$ (11)

The final representation *Z* ^*final*^ is input into the decoder to reconstruct the original similarity graph. The decoder is defined as Eq. 12 (Kipf and Welling, 2016b).

$$\hat{A}=\tau \left(Z^{\mathrm{fi}nal}{Z^{\mathrm{fi}nal}}^{T}\right)$$ (12)

After the neural network optimization is completed, a standard clustering method named K-means (Ding and He, 2004) is applied to the final representation *Z* ^*final*^ to identify cancer subtypes.

## 3 Experiments and Results

To evaluate the performance of our proposed-method multiGATAE, we compared it with eight state-of-the-art clustering methods, namely, DLSF (Zhang et al., 2022), subtype-WESLR (Song et al., 2021), SNF (Wang et al., 2014), NEMO (Rappoport and Shamir, 2019), iClusterBayes (Mo et al., 2018), moCluster (Meng et al., 2016), LRAcluster (Wu et al., 2015), and PFA (Shi et al., 2017) on eight public cancer multi-omics datasets. Here, we first introduce the details of these eight state-of-the-art methods, then we introduce the datasets used in this section and show the experiment results on these eight datasets.

• NEMO is a multi-omics clustering method based on the neighborhood. NEMO first constructs inter-patient similarity network for each omics and then integrates these networks into one network. Finally, the network is used for clustering.
• iClusterBayes adopts latent variables to capture the inherent structure of multi-omics datasets. The latent variable space is then used to identify cancer subtypes.
• moCluster investigates the joint patterns among multi-omics datasets. It uses multi-block multivariate analysis to define a set of latent variables and passes it to the clustering method to identify the cancer subtypes.
• LRAcluster discovers shared latent subspaces of the multi-omics data based on the integrative probabilistic model. The shared latent subspaces can be applied to identify subtypes.
• SNF is a network fusion method. It generates similarity networks for single-omics data and fuses these independent similarity networks into a combined network. This combined network can be used for cancer clustering.
• PFA is a pattern fusion analysis framework. It can capture intrinsic structure from multi-omics data for cancer clustering.
• subtype-WESLR uses a weighted ensemble strategy to fuse base clustering obtained by distinct methods as prior knowledge and maps each omics data into a common latent subspace. The common latent subspace is optimized iteratively to identify cancer subtypes.
• DLSF is a novel cancer clustering method based on deep neural network. It uses a cycle autoencoder which has a shared self-expressive layer to merge latent representation at each omics level into a fused representation at the multi-omics level. The fused representation can be used to identify cancer subtypes.

### 3.1 Data Set and Data Preprocessing

Eight TCGA cancer public datasets including kidney renal clear cell carcinoma (KIRC), breast invasive carcinoma (BRCA), colon adenocarcinoma (COAD), skin cutaneous melanoma (SKCM), lung squamous cell carcinoma (LUSC), glioblastoma multiforme (GBM), liver hepatocellular carcinoma (LIHC), and ovarian serous cystadenocarcinoma (OV) were used in this work. They were downloaded from TCGA (Cancer Genome Atlas Research Network, 2008), and each of them contains four types of data: miRNA expression, mRNA expression, DNA methylation, and clinical profiles. These three datasets are preprocessed by the following steps. Outlier removal is the first step. The features with missing values in more than 20% samples were deleted. Similarly, samples which have more than 20% features were removed. Finally, 206 samples in KIRC, 623 in BRCA, 214 in COAD, 439 in SKCM, 271 in GBM, 337 in LUSC, 404 in LIHC, and 290 in OV remained in this step. The next step is missing-data imputation. K nearest neighbor (Troyanskaya et al., 2001) imputation had been applied to impute the missing values. Finally, all of these datasets were normalized as Eq. 13:

$$\tilde{f}=\frac{f-E\left(f\right)}{\sqrt{\mathrm{Var}\left(f\right)}}$$ (13)

where *E*(*f*) is the mean of f, and Var(*f*) is the variance of f.

### 3.2 Optimal Number of Clusters

**FIGURE 2:**
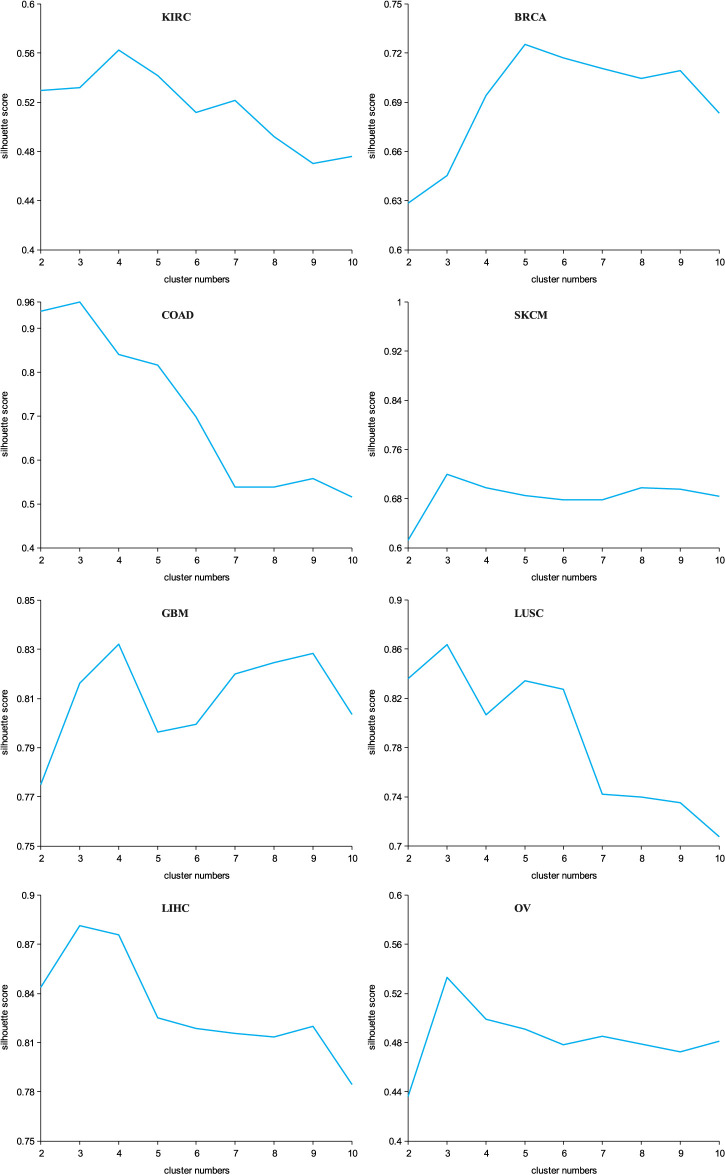
Silhouette width multiGATAE achieved on the eight datasets.

Since the K-means clustering method cannot automatically determine the optimal number of clusters, a silhouette width (Rand, 1971) was adopted to find the optimal clustering number. The parameters of our proposed method were also adjusted according to the silhouette width. We determined the optimal hidden layers, learning rate (Lr), and the dropout according to the grid search method. The optimal hidden layers were 2, Lr was 0.01, and dropout was 0.5, which achieved the best silhouette width and were finally applied in this work. In addition, for the compared methods, the parameters as given in their original articles were slightly modified to make them more suitable for our dataset. The silhouette width that our proposed method achieved on the eight datasets is shown in Figure 2.

Since the sample size of the cancer omics data is not very large, an excessive number of clusters may introduce bias. Thus, the number of clusters adopted in this work ranged from two to 10. The range of the silhouette width was from −1 to 1, and the closer it was to 1 meant the better the clustering performance was. We can see from Figure 2 that within a certain range, the silhouette width exhibited an increasing tendency. After reaching the optimal cluster number, the silhouette width started to gradually decrease. Specifically, for the KIRC datasets, the silhouette width achieved was the best when the cluster number was set to 4. This meant that the best clustering results were obtained when KIRC was clustered into four subtypes. Similarly, the BRCA was finally clustered into five subtypes, the COAD into three subtypes, the SKCM into three subtypes, the GBM into four subtypes, the LUSC into three subtypes, the LIHC into three subtypes, and the OV dataset into three subtypes. We can see that all the optimal numbers are within five, and this may indicate that the amount of available data was not sufficient to identify numerous cancer subtypes.

### 3.3 Comparison With Other Methods

To validate the performance of our proposed-method multiGATAE, we compared it with eight state-of-the-art methods on eight cancer datasets. Due to the lack of labels for the omics data, the negative log10 *p*-value and C-index of log-rank test were used as the metric. The log-rank test of the Cox regression (Hosmer and Lemeshow, 1999) is a statistical model and is used to assess the difference in survival profiles between subtypes. The *p*-value represents whether the observed differences are significant. If the *p*-value is less than 0.05, the observed subtypes are considered significantly different. To facilitate comparison, the negative and log operations were performed. The C-index was used to assess the predictive performance of the survival model. The results are shown in Table 1.

**TABLE 1:** Results of comparison methods and the proposed method, the first value is cluster number and the second is the negative log10 *p*-value.

Metric	Algorithm	KIRC	BRCA	COAD	SKCM	GBM	LUSC	LIHC	OV
*p*-value	NEMO	3/4.48	4/0.31	4/0.96	4/2.74	3/2.96	3/2.15	3/1.60	3/0.05
	iClusterBayes	4/2.51	5/1.06	4/0.09	4/1.85	3/0.22	3/1.24	3/1.11	3/1.48
	moCluster	3/2.82	5/3.31	3/1.04	4/2.98	3/1.96	3/2.31	2/1.02	3/1.60
	LRAcluster	3/2.07	5/2.23	4/1.17	3/3.25	3/2.00	3/2.35	3/0.39	3/2.96
	SNF	3/3.40	4/2.82	3/1.07	4/2.31	3/2.92	3/2.03	3/1.54	3/1.15
	PFA	2/2.08	5/2.89	3/1.00	4/2.64	2/2.23	3/1.04	2/2.64	3/0.05
	subtype-WESLR	4/4.76	**5/5.24**	4/2.43	5/5.00	3/3.84	5/2.30	**4/5.21**	3/3.44
	DLSF	4/2.76	3/1.89	4/0.05	5/3.85	**5/4.53**	3/0.11	3/3.15	4/0.03
	multiGATAE	**4/5.30**	5/1.68	**3/3.12**	**3/5.52**	4/4.0	**3/2.60**	3/3.51	**3/5.40**
C-index	NEMO	0.654	0.526	0.557	0.56	0.533	0.565	0.535	0.514
	iClusterBayes	0.617	0.535	0.552	0.542	0.515	0.516	0.557	0.536
	moCluster	0.626	0.588	0.543	0.566	0.538	0.576	0.553	0.56
	LRAcluster	0.597	0.539	0.579	0.562	0.551	0.572	0.541	0.584 2
	SNF	0.638	0.587	0.568	0.565	0.544	0.566	0.538	0.543
	PFA	0.581	0.544	0.57	0.564	0.538	0.52	0.555	0.567
	subtype-WESLR	**0.66**	0.595	0.632	0.58	0.559	0.587	0.594	0.581
	DLSF	0.623	**0.627**	0.539	0.578	0.582	0.527	0.575	0.563
	multiGATAE	0.618	0.574	**0.644**	**0.594**	**0.587**	**0.614**	**0.599**	**0.61**

Bold values indicates the best values.

**FIGURE 3:**
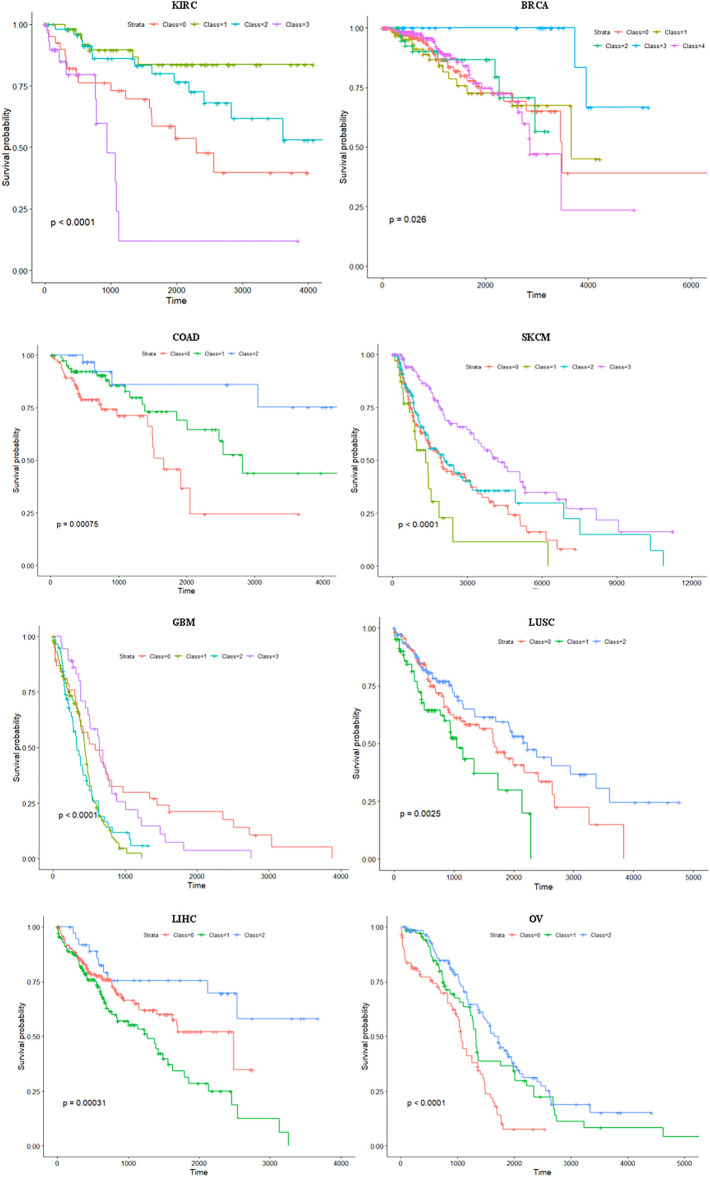
Survival curves for eight cancer datasets.

It can be seen from Table 1 that our proposed-method multiGATAE achieved the best performance on most datasets. Specifically, on the KIRC dataset, the negative log10 *p*-value that multiGATAE achieved was 5.30, which is 0.54 higher than the best remaining method subtype-WESLR. As for COAD, SKCM, LUSC, and OV datasets, the multiGATAE achieved 0.69, 0.52, 0.3, and 1.96 improvements compared with the best remaining method. As for the C-index, except for KIRC and BRCA, multiGATAE outperformed the compared methods on the other datasets. This demonstrates that the subtypes identified by our proposed method are indeed survival distinct. To illustrate the difference between the subtypes identified by our proposed method clearly, the survival curves for the eight cancer datasets are shown in Figure 3. As can be seen in Figure 3, except for BRCA, the cancer subtypes identified by our method on the other seven datasets all exhibit significantly different survival curves. The survival curve was significantly different between the subtypes, and this difference became progressively greater with time, indicating that the probability of survival varies between subtypes. For example, in the case of KRIC, subtype 3 showed a very low survival probability compared to the other subtypes when the time was above 1,000. This suggests that our method could identify groups of patients with different prognoses and help with precision treatment.

### 3.4 Analysis of Identified Subtypes on Lung Squamous Cell Carcinoma

**FIGURE 4:**
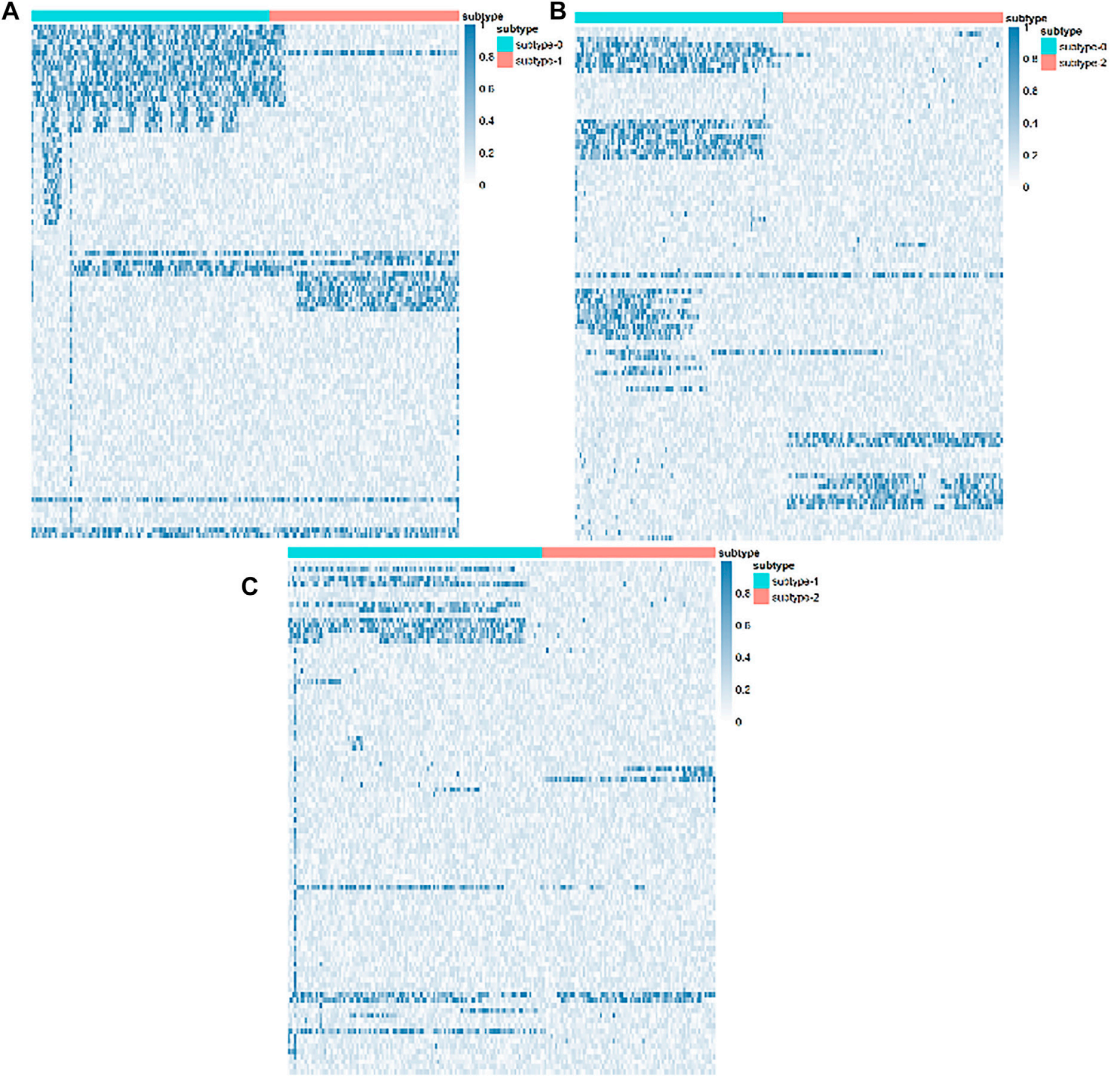
Differentially expressed mRNAs of the LUCS subtypes. **(A)** Subtype 0 and subtype 1. **(B)** Subtype 0 and subtype 2. **(C)** Subtype 1 and subtype 2.

In order to further validate our proposed method, we selected LUSC for a relevant biological analysis of identified subtypes. There were three subtypes identified by our proposed method, and in order to discover the differences at the molecular level between these three subtypes, we performed differential mRNA expressions by R package limma (Smyth, 2005). The differentially expressed mRNAs are shown by the heat map in Figure 4. As we can see from Figure 4, there are mRNAs which are significantly differentially expressed. This demonstrates that the subtypes identified by our proposed method have molecular-level differences.

### 3.5 Effectiveness of Multi-Omics Data

In this work, we used multi-omics data in order to obtain a comprehensive view on cancer subtype identification. To investigate the difference in results between single-omics and multi-omics data, we carried out experiments with single-omics data. The results are shown in Table 2. It can be seen from Table 2 that multiGATAE with multi-omics data performed better than using single-omics data. This suggests that integrating multi-omics data helps to capture a better embedded expression and thus identify more stable cancer subtypes. Besides, the DNA methylation data showed relatively better results compared with the other omics data. This may indicate that the DNA methylation data contains more information that facilitates cancer subtype identification.

**TABLE 2:** Results of multi-omics and single-omics, the first value is cluster number and the second is the negative log10 *p*-value.

	KIRC	BRCA	COAD	SKCM	GBM	LUSC	LIHC	OV
mRNA	4/1.31	3/0.20	3/0.24	3/1.52	4/1.27	3/0.38	3/0.8	3/0.97
DNA methylation	3/1.75	3/0.71	3/0.73	3/1.69	4/1.71	3/0.03	3/0.87	3/2.85
miRNA	4/1.57	4/0.39	3/0.98	3/1.98	4/1.24	4/0.53	3/0.667	3/1.35
Multi-omics	4/5.30	5/1.68	3/3.12	3/5.52	4/4.0	3/2.60	3/3.51	3/5.40

## 4 Conclusion

Cancer is a highly heterogeneous disease that causes a large number of deaths every year. Cancer subtype identification aims to identify groups of patients with different clinical outcomes for precise treatment. In this work, we proposed a novel cancer subtype identification method named multiGATAE. multiGATAE first constructed a similarity graph by integrating multi-omics data, and then input the similarity graph and the omics data into a graph autoencoder network which is composed of a graph attention network and an omics-level attention mechanism to obtain the embedding representation. Once gaining the embedding representation, the K-means clustering method was applied to it to identify subtypes. multiGATAE was compared with eight state-of-the-art methods on eight public cancer datasets. The results demonstrate that our proposed method can identify distinct subtypes with different survival outcomes. In the future, we consider integrating more data to develop our method. In addition, when learning embedding representation, taking clustering losses into consideration is also a way to improve our method.

## Data Availability

Publicly available datasets were analyzed in this study. This data can be found here: https://portal.gdc.cancer.gov/.

## References

[B1] Bass A. J. Thorsson V. Shmulevich I. Reynolds S. M. Miller M. Bernard B. (2014). Comprehensive Molecular Characterization of Gastric Adenocarcinoma. Nature 513, 202–209. 10.1038/nature13480 25079317 PMC4170219

[B2] Cancer Genome Atlas Research Network (2008). Comprehensive Genomic Characterization Defines Human Glioblastoma Genes and Core Pathways. Nature 455, 1061. 10.1038/nature07385 18772890 PMC2671642

[B3] Chaudhary K. Poirion O. B. Lu L. Garmire L. X. (2018). Deep Learning-Based Multi-Omics Integration Robustly Predicts Survival in Liver Cancer. Clin. Cancer Res. 24, 1248–1259. 10.1158/1078-0432.CCR-17-0853 28982688 PMC6050171

[B4] Ding C. He X. (2004). “K-means Clustering via Principal Component Analysis.” in Proceedings of the 21 st International Conference on Machine Learning, Banff, Canada, July 2004. 10.1145/1015330.1015408

[B5] Gomez-Cabrero D. Abugessaisa I. Maier D. Teschendorff A. Merkenschlager M. Gisel A. (2014). Data Integration in the Era of Omics: Current and Future Challenges. BMC Syst. Biol. 8, 1–10. 10.1186/1752-0509-8-S2-I1 25032990 PMC4101704

[B6] Hosmer D. W. Lemeshow S. (1999). Applied Survival Analysis: Time-To-Event, Vol. 317. Hoboken, New Jersey, United States: Wiley-Interscience.

[B7] Kipf T. N. Welling M. (2016a). Semi-supervised Classification with Graph Convolutional Networks. arXiv. arXiv preprint arXiv:1609.02907.

[B8] Kipf T. N. Welling M. (2016b). Variational Graph Auto-Encoders. arXiv. arXiv preprint arXiv:1611.07308.

[B9] Le Van T. Van Leeuwen M. Carolina Fierro A. De Maeyer D. Van den Eynden J. Verbeke L. (2016). Simultaneous Discovery of Cancer Subtypes and Subtype Features by Molecular Data Integration. Bioinformatics 32, i445–i454. 10.1093/bioinformatics/btw434 27587661

[B10] Liang C. Shang M. Luo J. (2021). Cancer Subtype Identification by Consensus Guided Graph Autoencoders. Bioinformatics 37, 4779–4786. 10.1093/bioinformatics/btab535 34289034

[B11] Meng C. Helm D. Frejno M. Kuster B. (2016). Mocluster: Identifying Joint Patterns across Multiple Omics Data Sets. J. proteome Res. 15, 755–765. 10.1021/acs.jproteome.5b00824 26653205

[B12] Mo Q. Shen R. Guo C. Vannucci M. Chan K. S. Hilsenbeck S. G. (2018). A Fully Bayesian Latent Variable Model for Integrative Clustering Analysis of Multi-type Omics Data. Biostatistics 19, 71–86. 10.1093/biostatistics/kxx017 28541380 PMC6455926

[B13] Rand W. M. (1971). Objective Criteria for the Evaluation of Clustering Methods. J. Am. Stat. Assoc. 66, 846–850. 10.1080/01621459.1971.10482356

[B14] Rappoport N. Shamir R. (2019). Nemo: Cancer Subtyping by Integration of Partial Multi-Omic Data. Bioinformatics 35, 3348–3356. 10.1093/bioinformatics/btz058 30698637 PMC6748715

[B15] Shen R. Olshen A. B. Ladanyi M. (2009). Integrative Clustering of Multiple Genomic Data Types Using a Joint Latent Variable Model with Application to Breast and Lung Cancer Subtype Analysis. Bioinformatics 25, 2906–2912. 10.1093/bioinformatics/btp543 19759197 PMC2800366

[B16] Shi Q. Zhang C. Peng M. Yu X. Zeng T. Liu J. (2017). Pattern Fusion Analysis by Adaptive Alignment of Multiple Heterogeneous Omics Data. Bioinformatics 33, 2706–2714. 10.1093/bioinformatics/btx176 28520848

[B17] Smyth G. K. (2005). Limma: Linear Models for Microarray Data. In Bioinformatics and Computational Biology Solutions Using R and Bioconductor. Berlin/Heidelberg, Germany: Springer, 397–420. 10.1007/0-387-29362-0_23

[B18] Sohn B. H. Hwang J.-E. Jang H.-J. Lee H.-S. Oh S. C. Shim J.-J. (2017). Clinical Significance of Four Molecular Subtypes of Gastric Cancer Identified by the Cancer Genome Atlas Project. Clin. Cancer Res. 23, 4441–4449. 10.1158/1078-0432.CCR-16-2211 28747339 PMC5785562

[B19] Song W. Wang W. Dai D.-Q. (2021). Subtype-WESLR: Identifying Cancer Subtype with Weighted Ensemble Sparse Latent Representation of Multi-View Data. Brief. Bioinform. 23, bbab398. 10.1093/bib/bbab398 34607358

[B20] Sung H. Ferlay J. Siegel R. L. Laversanne M. Soerjomataram I. Jemal A. (2021). Global Cancer Statistics 2020: Globocan Estimates of Incidence and Mortality Worldwide for 36 Cancers in 185 Countries. CA: a Cancer J. clinicians 71, 209–249. 10.3322/caac.21660 33538338

[B21] Troyanskaya O. Cantor M. Sherlock G. Brown P. Hastie T. Tibshirani R. (2001). Missing Value Estimation Methods for Dna Microarrays. Bioinformatics 17, 520–525. 10.1093/bioinformatics/17.6.520 11395428

[B22] Vaswani A. Shazeer N. Parmar N. Uszkoreit J. Jones L. Gomez A. N. (2017). “Attention Is All You Need,” in Advances in neural information processing systems, Vancouver, December 2004. Editors Saul L. K. Weiss Y. Bottou L., 5998–6008.

[B23] Veličković P. Cucurull G. Casanova A. Romero A. Lio P. Bengio Y. (2017). Graph Attention Networks. arXiv. arXiv preprint arXiv:1710.10903.

[B24] Wang B. Mezlini A. M. Demir F. Fiume M. Tu Z. Brudno M. (2014). Similarity Network Fusion for Aggregating Data Types on a Genomic Scale. Nat. Methods 11, 333. 10.1038/nmeth.2810 24464287

[B25] Wang C. Pan S. Hu R. Long G. Jiang J. Zhang C. (2019). “Attributed Graph Clustering: A Deep Attentional Embedding Approach,” in Proceedings of the 28th International Joint Conference on Artificial Intelligence, Macao China, August 2019. 10.24963/ijcai.2019/509

[B26] Wu D. Wang D. Zhang M. Q. Gu J. (2015). Fast Dimension Reduction and Integrative Clustering of Multi-Omics Data Using Low-Rank Approximation: Application to Cancer Molecular Classification. BMC genomics 16, 1022. 10.1186/s12864-015-2223-8 26626453 PMC4667498

[B27] Wu Z. Pan S. Chen F. Long G. Zhang C. Yu P. S. (2021). A Comprehensive Survey on Graph Neural Networks. IEEE Trans. Neural Networks Learn. Syst. 32, 4–24. 10.1109/TNNLS.2020.2978386 32217482

[B28] Xu A. Chen J. Peng H. Han G. Cai H. (2019). Simultaneous Interrogation of Cancer Omics to Identify Subtypes with Significant Clinical Differences. Front. Genet. 10, 236. 10.3389/fgene.2019.00236 30984238 PMC6448130

[B29] Yang B. Xin T.-T. Pang S.-M. Wang M. Wang Y.-J. (2021a). Deep Subspace Mutual Learning for Cancer Subtypes Prediction. Bioinformatics 37, 3715–3722. 10.1093/bioinformatics/btab625 34478501

[B30] Yang B. Zhang Y. Pang S. Shang X. Zhao X. Han M. (2021b). Integrating Multi-Omic Data with Deep Subspace Fusion Clustering for Cancer Subtype Prediction. IEEE/ACM Trans. Comput. Biol. Bioinform. 18, 216–226. 10.1109/TCBB.2019.2951413 31689204

[B31] Yang H. Chen R. Li D. Wang Z. (2021c). Subtype-GAN: a Deep Learning Approach for Integrative Cancer Subtyping of Multi-Omics Data. Bioinformatics 37, 2231–2237. 10.1093/bioinformatics/btab109 33599254

[B32] Zhang C. Chen Y. Zeng T. Zhang C. Chen L. (2022). Deep Latent Space Fusion for Adaptive Representation of Heterogeneous Multi-Omics Data. Brief. Bioinform., Bbab600. 10.1093/bib/bbab600 35079777

[B33] Zhao L. Lee V. H. Ng M. K. Yan H. Bijlsma M. F. (2019). Molecular Subtyping of Cancer: Current Status and Moving toward Clinical Applications. Brief. Bioinformatics 20, 572–584. 10.1093/bib/bby026 29659698

[B34] Zhao L. Yan H. (2019). Mcnf: A Novel Method for Cancer Subtyping by Integrating Multi-Omics and Clinical Data. IEEE/ACM Trans. Comput. Biol. Bioinformatics 17, 1682–1690. 10.1109/TCBB.2019.2910515 30990192

